# A Molecular Diagnostic Approach for Hypertension Through Establishment of a Metabolite Risk Score Using a Multi-Metabolite Panel Identified via UHPLC-MS/MS

**DOI:** 10.3390/ijms27188130

**Published:** 2026-09-12

**Authors:** Youngmin Han, Hye Jin Yoo

**Affiliations:** 1 School of Biomedical Health Science and Engineering, College of Engineering, University of Ulsan, Ulsan 44610, Republic of Korea; ymhan@ulsan.ac.kr; 2 Institute for Specialized Teaching and Research (INSTAR), Inha University, Incheon 22212, Republic of Korea; 3 BK21 FOUR Program in Biomedical Science and Engineering, Department of Biomedical Science, Inha University, Incheon 22212, Republic of Korea

**Keywords:** hypertension, metabolite risk score, metabolomics, molecular diagnosis, discriminative performance

## Abstract

Hypertension (HTN) is often asymptomatic and difficult to detect using blood pressure (BP) measurements unless BP is substantially elevated. Given its association with metabolic alterations and complications, this study aimed to establish a metabolite risk score (MRS) as a molecular tool to complement BP-based diagnosis. Plasma samples and clinical data from healthy individuals and HTN patients were obtained through the Korea Biobank Network, and non-targeted metabolomics was performed. Eight HTN-associated key metabolites were selected by least absolute shrinkage and selection operator (LASSO) regression. An MRS was calculated as their weighted sum in the discovery set and subsequently validated in the replication set. The MRS showed strong discriminative performance for HTN status in the replication set [area under the curve (AUC) = 0.926, 95% confidence interval (CI): 0.876–0.976] and remained significantly associated with prevalent HTN after adjustment for age and BMI [odds ratio (OR) = 1.747, 95% CI: 1.317–2.318, *p* < 0.001]. At the MRS cut-off, classification accuracy in the replication set was approximately 84%, with 83.7% sensitivity and 84% specificity. The MRS also showed weak-to-moderate positive correlations with systolic BP in both the discovery set (*r* = 0.320, *p* = 0.001) and the replication set (*r* = 0.335, *p* < 0.001); however, these correlations were no longer statistically significant after adjustment for age and BMI. These findings support further evaluation of the MRS as a complementary molecular approach for HTN discrimination. Integration with other omics platforms may facilitate the development of more comprehensive molecular approaches for HTN. Further validation in larger prospective longitudinal cohorts is required before its potential clinical application.

## 1. Introduction

Hypertension (HTN) represents a significant global public health challenge, with a high prevalence among adults worldwide [1]. In Korea, approximately 30% of the adult population, equivalent to nearly 13 million individuals, were estimated to have HTN as of 2022 [2]. It is widely recognized as a leading risk factor for disease, affecting cardiovascular [3] and kidney [4] functions and contributing to serious complications. Given these significant health risks, accurate and early diagnosis of HTN is critical. To promote cardiovascular disease prevention and early intervention, the American College of Cardiology and American Heart Association (ACC/AHA) have revised the diagnostic guidelines by lowering the threshold values for blood pressure (BP) in their 2017 guideline, and these thresholds have been maintained in the updated 2025 ACC/AHA guideline [5].

Despite its clinical importance, the current standard for HTN diagnosis is primarily based on BP measurements (in mmHg) [2]. BP measurement provides direct and clinically essential information; however, it does not characterize the molecular and metabolic alterations that may accompany HTN. Since HTN often develops gradually without obvious symptoms [6,7], characterization of such molecular changes may provide complementary information on the underlying disease phenotype. Although metabolic profiling likewise reflects the physiological state at a specific time point, it provides a different type of information by characterizing circulating molecular and metabolic alterations. In this context, metabolomic profiling may offer an additional molecular perspective alongside conventional BP assessment, rather than serving as a replacement for BP-based diagnosis. Furthermore, given that HTN is a major risk factor for a range of serious complications [8], a better understanding of its associated molecular alterations may provide additional insight into the broader metabolic characteristics of the disease.

Indeed, HTN is accompanied by interconnected pathophysiological alterations, including oxidative stress, chronic low-grade inflammation, endothelial dysfunction, neurohormonal dysregulation, and disturbances in lipid and energy metabolism [9,10,11]. These alterations can affect cellular energy utilization, mitochondrial function, and lipid and amino acid metabolism, thereby changing the production, utilization, and circulating levels of multiple molecules [10,11]. Consequently, individuals with HTN may exhibit metabolomic profiles that differ from those of healthy individuals. Consistent with this, previous metabolomic studies have reported alterations in amino acids, lipids, acylcarnitines, and other metabolic intermediates in association with HTN [12,13]. Such findings provide a biological basis for using circulating metabolomic profiles to characterize the molecular phenotype associated with HTN.

In this context, metabolites, which closely reflect phenotypic changes, are suitable as biomarkers for molecular assessment. Numerous studies have employed metabolomics to identify novel biomarkers associated with HTN and other diseases [12,14]. However, most of these efforts have focused on discovering a single statistically significant metabolite related to a specific phenotype of interest. Given the multifactorial nature of complex phenotypes such as HTN, it is improbable that a single biomarker can fully capture the underlying biological heterogeneity [3,15]. Consequently, an integrated multi-metabolite approach may be useful for enhancing diagnostic precision and advancing our mechanistic understanding of the disease.

Efforts to integrate multiple molecular markers for phenotypic prediction have been more extensively developed in the field of genomics. The genetic risk score (GRS) and polygenic risk score (PRS) represent a well-established framework that quantifies an individual’s genetic susceptibility to disease by aggregating the cumulative effects of multiple risk-associated genetic variants, each weighted according to its estimated effect size [16,17,18]. However, whereas GRS/PRS primarily reflect inherited genetic susceptibility, they do not directly characterize an individual’s current molecular and metabolic state. In this context, composite metabolite scores have been explored as an approach to integrate multiple circulating metabolite signals associated with disease phenotypes [19,20]. The present study therefore constructed a metabolite risk score (MRS) by integrating multiple metabolite levels into a weighted sum based on their relative contributions, with the aim of capturing HTN-associated metabolic alterations that may provide complementary molecular information alongside conventional clinical measures such as BP. In addition, as the MRS is not yet a well-established or standardized framework comparable to GRS/PRS, the present study may provide evidence regarding the potential utility of this approach.

Based on these considerations, the present study aims to identify a panel of metabolites associated with HTN through comprehensive metabolomics analysis, construct an MRS, and evaluate its discriminative performance for prevalent HTN compared with conventional BP measures, including systolic BP (SBP) and diastolic BP (DBP).

## 2. Results

### 2.1. Clinical and Biochemical Characteristics in the Healthy and HTN Groups of the Discovery Set

A total of 200 Korean individuals, comprising 100 healthy individuals and 100 HTN patients, were randomly divided equally into discovery and replication sets, with each set including 50 healthy and 50 HTN participants. Using untargeted plasma metabolomic profiling, the discovery set was used to identify HTN-associated metabolites and construct the MRS, whereas the resulting MRS was subsequently evaluated in the replication set.

In the discovery set, none were excluded from the analysis. The group characteristics were confirmed clinically and biochemically, as the study participants were randomly selected by the Biobanks as described previously. The results are shown in Table 1. The HTN group was significantly older and had a higher body mass index (BMI), so age and BMI were used as confounding factors when adjusting for other variables. SBP and DBP and the levels of glucose, aspartate aminotransferase (AST), and alanine aminotransferase (ALT) were significantly higher in the HTN group both before and after the adjustment. Levels of low-density lipoprotein (LDL)-cholesterol and gamma-glutamyl transpeptidase (γ-GTP) were significantly increased in the HTN group after adjusting for the confounding factors, whereas the significance of total cholesterol levels disappeared after the adjustment.

### 2.2. Distinguished Metabolite Characteristics Between the Groups in the Discovery Set

Initial feature detection and quality filtering were performed using a minimum peak intensity of 500,000 and a peak-rating threshold of 5 in at least 10 input files, resulting in 306 and 214 features in the ESI positive and negative modes, respectively. After excluding unknown features and annotations with insufficient library support, 35 and 36 putatively annotated metabolites remained in the ESI positive and negative modes, respectively. Following the exclusion of drug-related compounds, 23 putatively annotated metabolites were retained in the ESI positive mode and 30 in the ESI negative mode.

**Figure 1:**
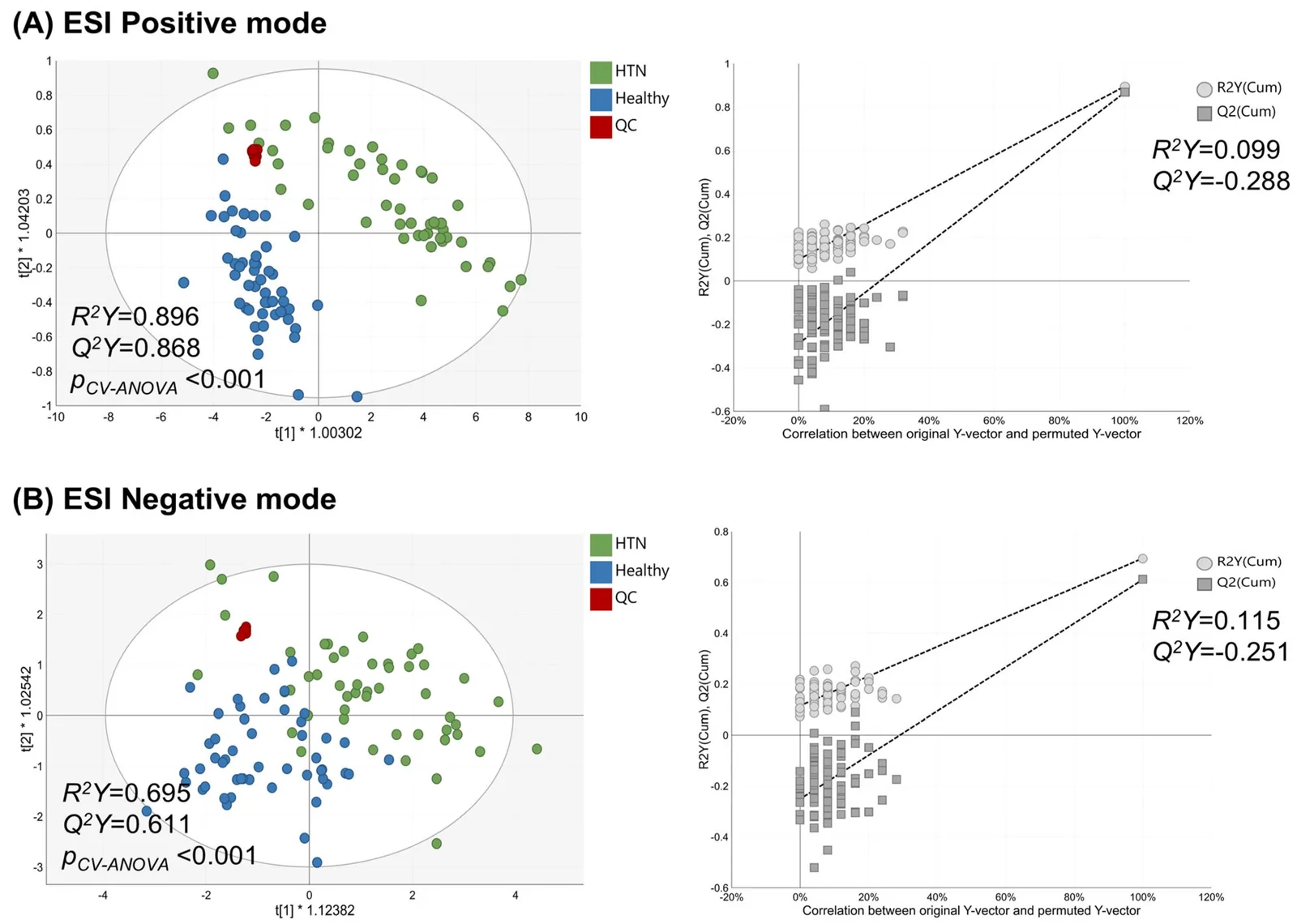
**OPLS-DA results showing group separation based on metabolite patterns in the discovery set and their permutation tests.** (**A**) Group separation in ESI positive mode (left) and the permutation test (100 times). (**B**) Group separation in ESI negative mode (left) and the permutation test (100 times).

To first examine the overall structure and sample distribution of the metabolomic data without using group information, unsupervised principal component analysis (PCA) was performed (Figure S1). The first two principal components explained 61.3% of the total variance in the ESI positive mode (PC1 = 53.1%, PC2 = 8.2%) and 52.5% in the ESI negative mode (PC1 = 40.2%, PC2 = 12.3%). Pooled QC samples clustered closely, while HTN samples showed a broader distribution than healthy samples in both ionization modes. Subsequently, supervised orthogonal partial least squares-discriminant analysis (OPLS-DA) was performed to evaluate class discrimination between the healthy and HTN groups based on their metabolite profiles (Figure 1). The metabolite profiles obtained from both ESI positive and negative modes significantly separated the healthy and HTN groups, as both *R^2^Y* and *Q*^2^*Y* values―indicating goodness of fit and predictive ability, respectively―exceeded 0.5 (ESI positive mode: *R*^2^*Y* = 0.896, *Q*^2^*Y* = 0.868, *p_CV-ANOVA_* < 0.001; ESI negative mode: *R*^2^*Y* = 0.695, *Q*^2^*Y* = 0.611, *p_CV-ANOVA_* < 0.001). To assess potential overfitting, 100 permutation tests were conducted by randomly shuffling the class labels. In the permutation plot of each ESI mode, the original model (at the far right) showed higher *R*^2^*Y* and *Q*^2^*Y* values than the models generated by random label permutations. This suggests that the observed class separation was unlikely to be attributable to random class assignment, and the negative *Q*^2^*Y* intercept further supports the absence of substantial overfitting (ESI positive mode: *R^2^Y* intercept = 0.099, *Q^2^Y* intercept = −0.288; ESI negative mode: *R^2^Y* intercept = 0.115, *Q^2^Y* intercept = −0.251) (Figure 1). Representative total ion chromatograms (TICs) of a pooled QC sample showed no apparent abnormalities in the chromatographic signal profiles in either ESI positive or negative mode (Figure S2). In addition, the median full width at half maximum (FWHM) of the chromatographic peaks corresponding to the detected compounds in the pooled QC sample was 0.068 min [interquartile range (IQR), 0.055–0.090 min] in ESI positive mode and 0.072 min (IQR, 0.063–0.088 min) in ESI negative mode. These generally narrow peak widths are favorable for chromatographic resolution.

### 2.3. Multi-Metabolite Panel for Hypertension

**Figure 2:**
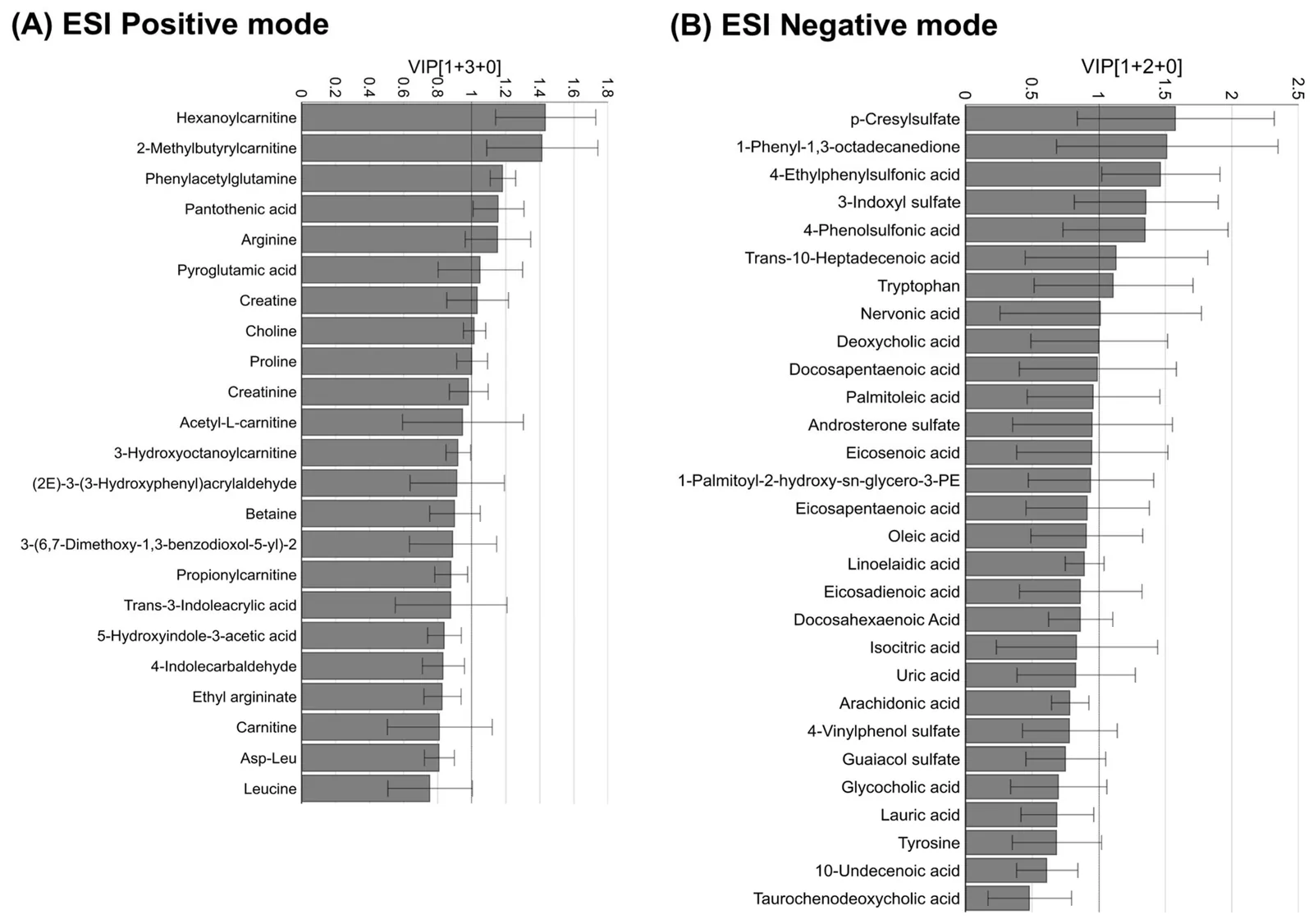
**VIP score analysis in the discovery set.** (**A**) ESI positive mode. Top nine metabolites showed a VIP score above 1. (**B**) ESI negative mode. Top nine metabolites showed a VIP score above 1. A total of the eighteen metabolites (VIP ≥1 in both ESI positive and negative modes) had an FDR-adjusted *p* value of <0.05, indicating statistically significant differences between the healthy and hypertension groups (major metabolites).

As shown in Figure 2, VIP values were obtained to identify the major metabolites that contribute to the discrimination between the healthy and HTN groups in the discovery set. In the ESI positive mode, nine metabolites had VIP values ≥ 1. In the ESI negative mode, another nine metabolites also had VIP values ≥ 1. The levels of the 18 major metabolites (with VIP values ≥ 1 in both ESI positive and negative modes) showed significant differences between the groups, as indicated by FDR-adjusted *p*-values less than 0.05 (Table S1).

To select key metabolites for establishing the MRS, LASSO regression analysis was conducted using the major metabolites identified above. As a result, an 8-metabolite panel for HTN was determined, comprising 2-methylbutyrylcarnitine, phenylacetylglutamine, 1-phenyl-1,3-octadecanedione, 4-phenolsulfonic acid, tryptophan, deoxycholic acid, nervonic acid, and p-cresylsulfate (Table 2). The LASSO coefficient values were used as weights in the subsequent MRS calculation.

### 2.4. Improved Diagnostic Performance of the MRS for Hypertension and Its Correlation with Conventional BP Measures

**Figure 3:**
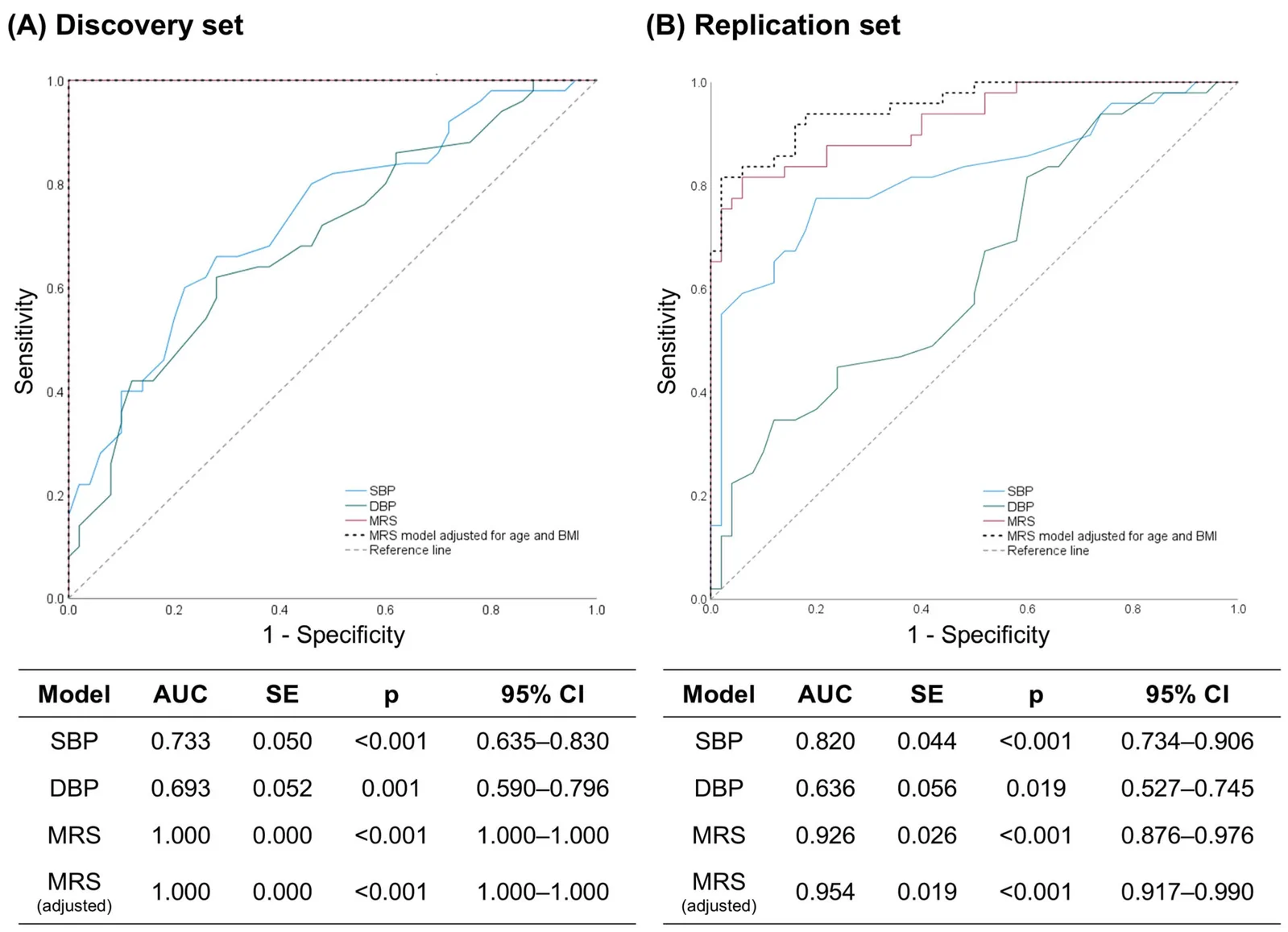
**Discriminatory performance of the MRS compared with the conventional blood pressure measures for prevalent hypertension.** The mean and standard deviation used to calculate *z*-scores, along with the weights used for the MRS calculation, were all obtained from the discovery set. These values were locked down and subsequently applied to the replication set for MRS calculation. An age- and BMI-adjusted MRS model was additionally evaluated. (**A**) Discriminatory performance of the MRS in the discovery set. (**B**) Evaluation of MRS discriminatory performance in the replication set.

In the discovery set, the MRS showed training performance with an AUC of 1.00 (*p* < 0.001) for distinguishing participants with HTN from healthy individuals, whereas the corresponding AUCs for conventional BP measures were 0.733 for SBP (*p* < 0.001) and 0.693 for DBP (*p* = 0.001) (Figure 3A). The MRS model adjusted for age and BMI also showed an AUC of 1.00 (*p* < 0.001) in the discovery set, with the training discrimination remaining unchanged after accounting for age and BMI (Figure 3A). In the replication set, the MRS showed high performance for distinguishing participants with HTN from healthy individuals (AUC = 0.926, *p* < 0.001), compared with SBP (AUC = 0.820, *p* < 0.001) and DBP (AUC = 0.636, *p* = 0.019) (Figure 3B). The model including MRS, age, and BMI showed a slightly higher AUC of 0.954 (*p* < 0.001), indicating that high discriminative performance was retained after accounting for age and BMI (Figure 3B). Moreover, logistic regression demonstrated that the MRS remained significantly associated with HTN status after adjustment for age and BMI in the replication set (Table S2).

Potential confounding by comorbid conditions was further assessed through an exploratory sensitivity analysis comparing HTN participants without recorded comorbid disease histories (*n* = 13) with healthy individuals (*n* = 50) in the replication set. As shown in Figure S3, the MRS retained high discriminative performance for HTN (AUC = 0.960, *p* < 0.001), compared with SBP (AUC = 0.892, *p* < 0.001) and DBP (AUC = 0.701, *p* = 0.027). The model including MRS, age, and BMI showed an AUC of 0.966 (*p* < 0.001). Consistent with the logistic regression results above, the MRS was significantly associated with HTN after adjustment for age and BMI in the sensitivity analysis (Table S2).

**Figure 4:**
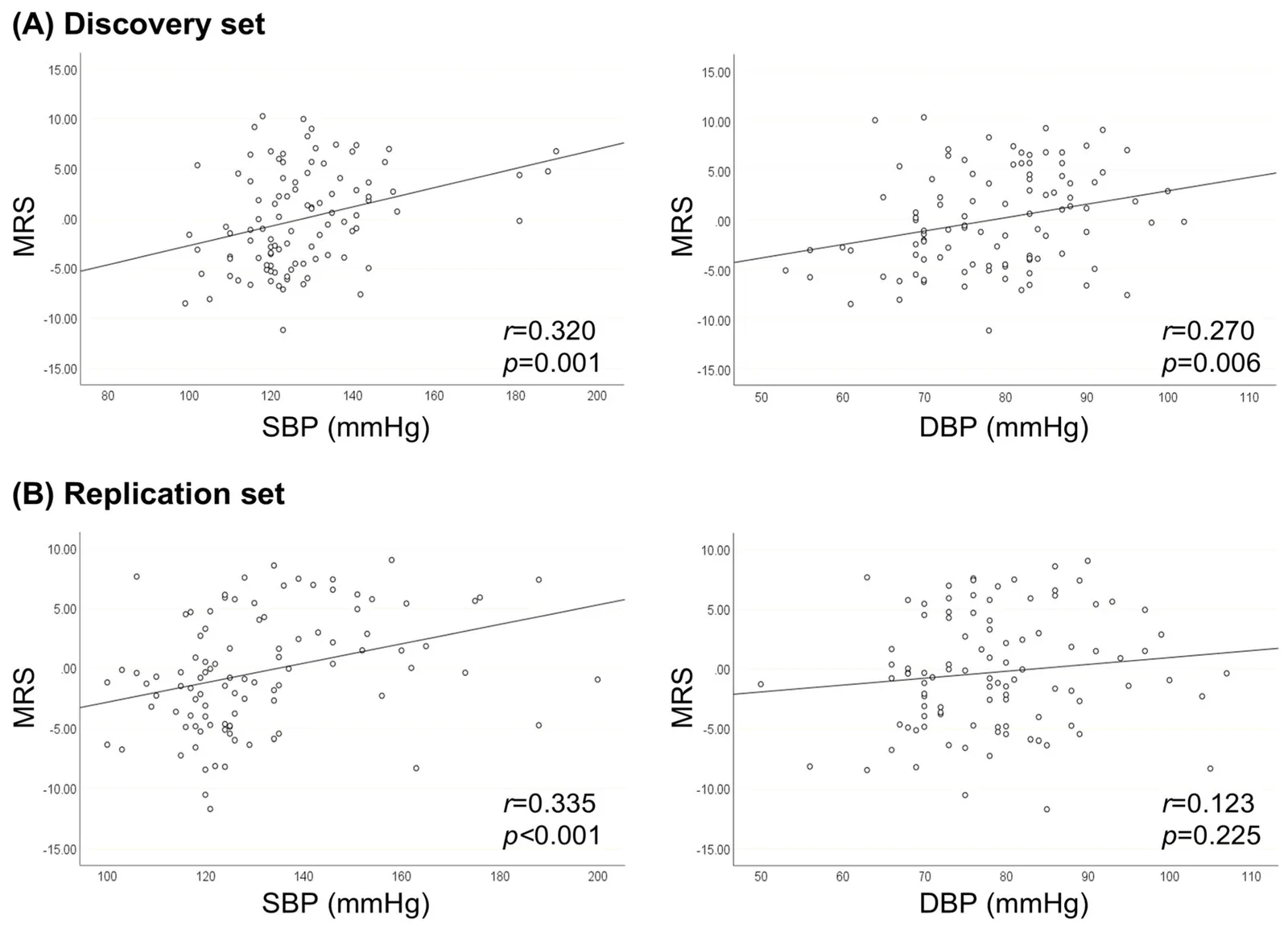
**Correlations between MRS and blood pressures in the discovery and replication sets.** (**A**) Scatter plots and correlation coefficients (*r*) between MRS and blood pressures (SBP and DBP) in whole individuals in the discovery set. (**B**) Scatter plots and correlation coefficients (*r*) between MRS and blood pressures (SBP and DBP) in whole individuals in the replication set.

To examine the relationship between the MRS and BP measures, correlations of the MRS with SBP and DBP were assessed in both the discovery and replication sets. As expected, the MRS showed significant positive correlations with both SBP and DBP in the discovery set (SBP: *r* = 0.320, *p* = 0.001; DBP: *r* = 0.270, *p* = 0.006) (Figure 4A). However, in the replication set, the MRS was significantly correlated with SBP only (*r* = 0.335, *p* < 0.001), but not with DBP (Figure 4B). After adjustment for age and BMI using partial correlation analysis, the correlations of the MRS with both SBP and DBP remained significant in the discovery set (Figure S4A). In the replication set, however, neither SBP nor DBP showed significant correlations with the MRS, although both associations were positive in direction (Figure S4B).

### 2.5. High Concordance Between MRS Cut-Off-Based Re-Grouping and Original Diagnostic Categories in the Replication Set

An MRS cut-off value of −0.431 was derived from the discovery set and subsequently applied unchanged to the replication set to evaluate its classification performance. Based on this predefined cut-off, individuals in the replication set were reclassified into two groups, healthy*_re-group_* and HTN*_re-group_*, as described in the Methods. With the new criterion, 41 out of 49 individuals originally diagnosed with HTN in the replication set were reclassified into HTN*_re-group_*, yielding an accuracy of 83.7%. The healthy*_re-group_* also showed a comparable reclassification accuracy, with 42 out of 50 originally healthy individuals correctly reclassified into the healthy*_re-group_*, resulting in an accuracy of 84.0%. Overall, the MRS cut-off demonstrated reasonable classification performance (Table 3).

### 2.6. Consistent and Significant Association of Elevated MRS with Dyslipidemia History in the Overall Study Population

**Figure 5:**
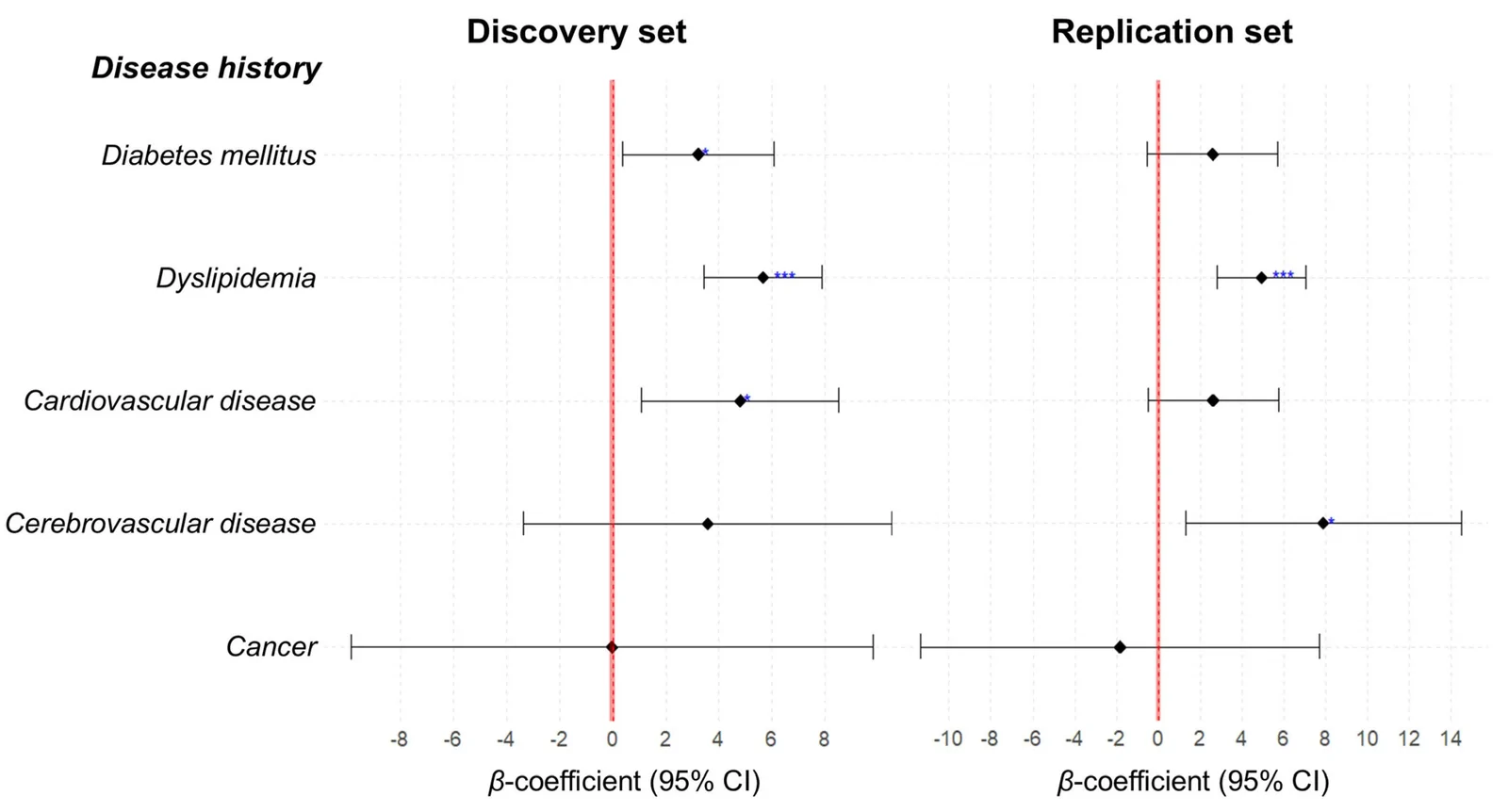
**Impact of disease history on MRS Association between disease history and MRS**. Dyslipidemia was significantly associated with increased MRS in both the discovery and replication sets, suggesting that elevated MRS may indicate the presence of dyslipidemia as a common comorbidity in hypertensive individuals. * *p* <0.05 and *** *p* <0.001.

As HTN is linked to other chronic diseases and cardiovascular events, the association between MRS and participants’ disease history was examined. In the discovery set, individuals with a history of diabetes mellitus, dyslipidemia, and cardiovascular disease had significantly higher MRS values by 3.22, 5.67, and 4.80, respectively (*p* = 0.029, *p* < 0.001, and *p* = 0.013, respectively) (Figure 5). In the replication set, the associations of MRS with histories of diabetes mellitus and cardiovascular disease were no longer significant, whereas a history of dyslipidemia remained significantly associated with a higher MRS of 4.95 (*p* < 0.001) (Figure 5). A history of cerebrovascular disease was also associated with an elevated MRS (7.91, *p* = 0.021) in the replication set (Figure 5). Among the examined comorbidities, dyslipidemia was the only condition consistently associated with higher MRS values in both sets.

Associations of SBP and DBP with the same disease-history variables were also examined as exploratory analyses (Figure S5). In the discovery set, neither SBP nor DBP was significantly associated with any of the disease histories (Figure S5A). In the replication set, a history of cerebrovascular disease was significantly associated with higher SBP, whereas no significant associations were observed between SBP and the other disease histories. DBP showed no significant association with any of the tested disease histories (Figure S5B).

## 3. Discussion

In the present study, unsupervised PCA revealed a broader distribution of metabolomic profiles within the HTN group than within the healthy group, which may reflect greater interindividual metabolic heterogeneity among participants with HTN. This heterogeneity may partly reflect the study design, as HTN participants were defined based on the KCD diagnosis code without further stratification according to recorded comorbid disease history or other HTN-related clinical characteristics, while detailed medication information was unavailable. Despite this heterogeneity, supervised OPLS-DA identified metabolomic variation associated with HTN status in the discovery set. The subsequent MRS, constructed from metabolites selected in the discovery set, retained high discriminative performance in the replication set, suggesting that the MRS captured HTN-associated metabolic information despite the broader heterogeneity observed within the HTN group. In the replication set, the MRS demonstrated significantly greater discriminative performance for HTN than SBP and DBP. The association between the MRS and prevalent HTN remained significant after adjustment for age and BMI. The MRS also showed a significant positive correlation with SBP in the replication set, although this correlation was attenuated after adjustment for age and BMI. In exploratory analyses of HTN-related comorbid disease history, dyslipidemia was consistently associated with higher MRS values in both the discovery and replication sets. Taken together, these findings support further evaluation of the MRS as a complementary molecular tool for assessment of prevalent HTN, while its clinical utility remains to be established.

Previous studies have demonstrated the utility of MRS across various diseases, supporting the validity of our analytical approach. MRS models have been used to predict various metabolic diseases [19,21,22]. Although studies specifically applying MRS to HTN are limited, some have reported associations between metabolic profiles and BP traits [19,23]. However, these studies often focus on individual metabolites or specific BP components, limiting their capacity to capture the complex, multifactorial nature of HTN. This study advances the field by constructing an MRS model that comprehensively incorporates HTN-related traits, thereby aiming to reflect the broader metabolic signature. Importantly, we employed LASSO regression, which is particularly well suited for high-dimensional omics data due to its combined feature selection and regularization capabilities [24]. This approach is particularly effective in managing multicollinearity and high dimensionality, both of which are common in untargeted metabolomics [25]. By shrinking irrelevant coefficients to zero, LASSO minimizes overfitting and yields a concise, interpretable set of predictive metabolites [26]. By integrating the resulting coefficients into a weighted *z*-score model, we incorporated the relative contribution of each selected metabolite to the fitted LASSO model into the MRS. Overall, our approach provides a basis for further development of an MRS framework and supports further evaluation of its association with HTN.

Among the eight metabolites selected through LASSO regression as components of the MRS, three (2-methylbutyrylcarnitine, 1-phenyl-1,3-octadecanedione, and tryptophan) showed relatively large absolute coefficients (|*β*| ≥ 1), reflecting their greater weighting in the overall MRS. Reduced levels of 2-methylbutyrylcarnitine, a short-chain acylcarnitine involved in branched-chain amino acid catabolism, have previously been associated with mitochondrial stress [27]. Mitochondrial dysfunction can promote oxidative stress and vascular dysfunction, which are implicated in vascular inflammation and HTN [9,28]. In the present study, 2-methylbutyrylcarnitine was also reduced in HTN patients and had a negative coefficient in the MRS, suggesting that its reduction may be associated with mitochondrial metabolic alterations in HTN. 1-Phenyl-1,3-octadecanedione exhibited the highest positive coefficient in our model. However, direct biological and functional evidence linking this metabolite to HTN is currently limited. A recent human metabolomics study reported increased levels of this metabolite in lung adenocarcinoma tissue [29]. In our study, levels of 1-phenyl-1,3-octadecanedione were also increased in HTN patients, showing a similar direction of change across different disease states. As the present study does not establish the physiological mechanisms underlying its association with HTN, the result should be interpreted as an observational finding rather than evidence of a mechanistic role in HTN. Tryptophan, an essential amino acid, plays a key role in immune regulation and vascular tone via the kynurenine pathway [30,31]. In the present study, tryptophan levels were reduced in HTN patients and had a negative coefficient in the MRS. Previous studies have reported that lower circulating tryptophan levels may reflect enhanced tryptophan catabolism under inflammatory conditions, which are often present in HTN and cardiovascular disease (CVD) [21,32]. Taken together with these previous reports, the reduced tryptophan levels observed in our HTN patients may suggest altered tryptophan metabolism. Overall, these metabolites contributed relatively large weights to the MRS, although the biological relevance of some of them to HTN requires further investigation. Moreover, because the metabolites identified in the present study remain putatively annotated, potential annotation ambiguity and in-source artifacts should be considered when interpreting their biological significance.

Because BP measurements are used to diagnose HTN, we also examined the relationship between the MRS and BP measures. In the present study, MRS was positively correlated with SBP in the replication set, whereas the correlation with DBP was not replicated. However, the MRS–SBP correlation was attenuated after adjustment for age and BMI, with statistical significance no longer observed. In contrast, MRS remained significantly associated with prevalent HTN after adjustment for age and BMI, and high discriminative performance was retained in the model including MRS, age, and BMI. These results suggest that the discriminatory ability of the MRS for prevalent HTN may not simply reflect the magnitude of BP elevation. Accordingly, a higher MRS may suggest that an individual’s metabolite profile more closely resembles the HTN-associated pattern identified by the model, rather than directly indicating BP level or HTN severity.

In addition to evaluating the discriminative performance of the MRS for HTN, the present study explored its associations with comorbid disease history, given that HTN frequently coexists with cardiometabolic conditions that may share metabolic abnormalities. Dyslipidemia was consistently and significantly associated with higher MRS values in both the discovery and replication sets. Given the frequent co-occurrence and metabolic overlap of HTN and dyslipidemia in clinical settings [13,33], the HTN-derived MRS may include metabolic features that are also associated with dyslipidemia. However, the present cross-sectional analysis cannot determine whether the observed MRS–dyslipidemia association reflects shared pathophysiological mechanisms, nonspecific metabolic overlap, or the co-occurrence of the two conditions. Future studies in larger and clinically diverse cohorts are therefore needed to determine whether the observed association with dyslipidemia is reproducible and whether associations between the MRS and comorbid disease history extend beyond dyslipidemia to other HTN-related comorbid conditions.

At the same time, the association between the MRS and dyslipidemia raises the question of whether the discriminative performance of the MRS for HTN may be influenced by coexisting dyslipidemia. To further examine this possibility, we performed a sensitivity analysis after excluding HTN participants with recorded comorbid disease histories. The MRS retained high discriminative performance in this sensitivity analysis, suggesting that its discrimination of HTN was not solely attributable to recorded comorbid conditions. Although the present sensitivity analysis provides additional evidence regarding the potential influence of comorbidities, further evaluation using separate disease-control groups, particularly individuals with dyslipidemia without HTN, is needed to determine the specificity of the MRS for HTN.

This study has several limitations. The findings are based on cross-sectional data; therefore, the MRS in the present study demonstrated an association with prevalent HTN rather than a validated predictive or prognostic capability. Whether the MRS can anticipate the future onset of HTN or HTN-related comorbidities requires evaluation in prospective longitudinal cohorts. Next, age and BMI differed between the healthy and HTN groups. To capture overall metabolomic differences associated with HTN status, these factors were not included as covariates in the initial metabolite selection process. Therefore, it remains uncertain whether the same metabolite panel would have been selected after accounting for age and BMI during feature selection. However, the resulting MRS was subsequently evaluated after adjusting for age and BMI, and its association with prevalent HTN remained statistically significant. In addition, information on antihypertensive and other relevant medication use was unavailable; therefore, potential medication-related effects on BP levels, circulating metabolomic profiles, and the association between MRS and HTN could not be accounted for. Finally, metabolites were putatively identified based on UHPLC-MS/MS data rather than confirmed with authentic reference standards; therefore, some uncertainty in metabolite identity remains and should be considered. Overall, these limitations highlight the need for further validation of the MRS in larger prospective longitudinal cohorts before its potential clinical application as a complementary molecular approach for HTN.

## 4. Materials and Methods

### 4.1. Sample Collection and Study Design

A total of 200 fasting plasma samples and corresponding clinical data were collected from the Biobank of Ajou University Hospital and the Biobank of Korea-Chungbuk National University Hospital (CBNUH), both of which are members of the Korea Biobank Network (KBN). KBN provides integrated guidelines to participating biobanks to support standardized biospecimen management and quality control, addressing pre-analytical factors such as plasma processing, centrifugation, storage containers, and storage conditions. The samples and data were provided for this study under a protocol reviewed by the Inha University Institutional Review Board (IRB) and determined to be exempt from ethical review (approval no./date: 21004-1AR/1 February 2023).

The 200 samples consisted of 100 healthy individuals and 100 HTN patients. These were randomly selected by the Biobanks according to the following inclusion criteria: (1) Korean adults aged 20 to 60 years; (2) Korean Standard Classification of Disease (KCD) code Z00 for healthy individuals; and (3) KCD code I10 for HTN patients. Each group (*n* = 100) was further randomly divided into discovery and replication sets at a 1:1 ratio, reflecting the two-phase cross-sectional design of the present study: phase 1 (discovery: discovery set, *n* = 50 per group) for identifying HTN-associated metabolites and constructing the MRS, and phase 2 (validation: replication set, *n* = 50 per group) for evaluating its performance. Note that one patient in the HTN group of the replication set was excluded due to poor sample quality during analysis; thus, the final number of subjects in that group was 49. The replication set was additionally used for an exploratory sensitivity analysis, in which HTN patients with recorded non-HTN comorbid disease histories were excluded. The resulting sensitivity set, derived from the replication set, consisted of 50 healthy individuals and 13 HTN participants without recorded disease histories.

Regarding the clinical data, the available variables included age, sex, BMI, SBP, DBP, glucose and lipid profiles, liver enzymes, and recorded disease histories, including diabetes mellitus, dyslipidemia, cardiovascular disease, cerebrovascular disease, and cancer. However, information on antihypertensive and other relevant medication use was not available.

### 4.2. Plasma Metabolite Profiling (Non-Targeted Metabolomics)

#### 4.2.1. Chemicals and Reagents

Acetonitrile (J.T.Baker^®^; Avantor, Radnor, PA, USA), methanol (J.T.Baker^®^; Avantor, Radnor, PA, USA), water (Thermo Fisher Scientific, Fair Lawn, NJ, USA) and formic acid (Thermo Fisher Scientific, Fair Lawn, NJ, USA), all of high purity and suitable for liquid chromatography–mass spectrometry (LC-MS) analysis, were used for plasma sample preparation and as mobile-phase components. The internal standards L-leucine-^13^C_6_ and stearic-d_35_ acid, both purchased from Sigma-Aldrich (St. Louis, MO, USA), were used during sample preparation. Suppliers for these reagents are cited here and are not repeated elsewhere in this section.

#### 4.2.2. Sample Preparation

The plasma sample (100 μL) was used for the ultra-high-performance liquid chromatography–tandem mass spectrometry (UHPLC-MS/MS). Cold acetonitrile (70%, 300 μL) was added to the plasma samples and vortexed, and then the samples were incubated at 4 °C for 10 min. The mixtures were subsequently centrifuged (13,000 rpm, 4 °C, 15 min). The supernatant was transferred to new microtubes and dried using a SpeedVac (Gyrozen, Gimpo, Gyeonggi-do, Republic of Korea). The residues were re-suspended in 10% cold methanol (100 μL) containing two internal standards (ISTDs), L-leucine-^13^C_6_ and stearic-d_35_ acid. All samples were pooled to make quality control (QC) samples and prepared with the same protocol as described.

#### 4.2.3. UHPLC-MS/MS

Metabolic profiling of the prepared samples was performed using an Ultimate 3000 RSLC System (Thermo Fisher Scientific, Bremen, Germany) equipped with an Acquity UPLC BEH C18 column (2.1 mm × 100 mm, 1.7 μm; Waters, Milford, MA, USA). Each sample (5 μL) was injected into the system, and QC samples were placed every 10th sample in the sequence. Metabolites were separated on the column, the temperature of which was maintained at 50 °C. Mobile phases A and B were prepared as 0.1% formic acid in water and 0.1% formic acid in methanol, respectively (gradient: 0–100% B over 17 min at 0.4 mL/min). Electrospray ionization (ESI) positive and negative modes were applied in MS/MS analysis (Q-Exactive Orbitrap Plus; Thermo Fisher Scientific, Waltham, MA, USA). Metabolic profile data were acquired in full MS-ddMS^2^ over a mass-to-charge (*m*/*z*) range of 80~1000. MS parameters included a spray voltage of 3.5 kV, sheath gas at 50 a.u., auxiliary gas at 13 a.u., S-lens radio frequency level at 55, and capillary temperature at 370 °C.

#### 4.2.4. Putative Identification of Metabolites

Putative metabolite identification was performed using Compound Discoverer (version 3.3 SP 2; Thermo Fisher, Waltham, MA, USA). Candidate annotations were evaluated using four annotation sources within Compound Discoverer: Predicted Compound, mzCloud Search, Metabolika Search, and ChemSpider Search. Annotations were retained when supported by an mzCloud spectral match or when concordant annotations were obtained from at least two of the other annotation sources. Accurate-mass matching was performed using a mass tolerance of 5 ppm, and mzCloud MS/MS spectral matching was performed using the default match factor threshold of 50. As no authentic reference standards were analyzed in parallel with the study samples, the reported metabolite identities were considered putative rather than confirmed identifications.

#### 4.2.5. Multi-Metabolite Panel Selection and MRS Calculation

After identifying the metabolic profiles, metabolites with a variable importance in projection (VIP) value ≥ 1.00 and false discovery rate (FDR)-adjusted *p*-value (*q*-value) < 0.05 were initially selected as candidate metabolites. These were subjected to the least absolute shrinkage and selection operator (LASSO) regression to further screen key metabolites for constructing the MRS.

The MRS was calculated as a weighted sum of the key metabolites, expressed as ∑*β_i_*M*_i_*, where *β_i_* (LASSO-derived coefficient) represents the weight of each key metabolite and M*_i_* is its corresponding *z*-score. To ensure consistency, the *β_i_* values, predefined in the discovery set, were applied to the replication set, and *z*-scores in the replication set were calculated using the mean and standard deviation (SD) derived from the discovery set.

#### 4.2.6. Re-Grouping of the Replication Set Based on MRS Cut-Off

The MRS cut-off value was determined from the discovery set using receiver-operating characteristic (ROC) curve analysis with Youden’s index. This cut-off was then applied to the replication set as a diagnostic threshold for HTN: individuals with MRS values < cut-off were re-grouped as healthy (healthy*_re-group_*), and those with MRS ≥ cut-off were re-grouped as HTN (HTN*_re-group_*).

#### 4.2.7. Association Between MRS and Disease History

Given the associations between HTN and its related complications, the present study further examined whether the MRS was associated with participants’ history of related diseases, including diabetes mellitus, dyslipidemia, cardiovascular disease, cerebrovascular disease, and cancer. For comparison, the associations between SBP and DBP and disease history were also examined in both the discovery and replication sets. These associations were visualized using a forest plot.

#### 4.2.8. Statistical Analysis

OPLS-DA, permutation tests, and VIP value acquisition were conducted using SIMCA^®^ (version 17; Sartorius-Umetrics, Göttingen, Germany). FDR adjustment of *p*-values for metabolites, LASSO regression for key metabolite selection, and linear regression for forest plot analysis were performed using R (version 4.4.2; R Foundation for Statistical Computing, Vienna, Austria) with the packages fdrtool (version 1.2.18), glmnet (version 4.1.8), and ggplot2 (version 3.5.1), respectively. General statistical analyses, including group comparisons of clinical characteristics, ROC curve analyses to compare the discriminative performance of the MRS, SBP, DBP, and a multivariable model including MRS, age, and BMI, logistic regression analyses to evaluate the association between MRS and HTN status before and after adjustment for age and BMI, and Pearson and age- and BMI-adjusted partial correlation analyses with corresponding scatter plots, were performed using SPSS (version 28; IBM Corp., Armonk, NY, USA). To assess the potential influence of comorbid disease history, the ROC curve and logistic regression analyses were repeated in the sensitivity set derived from the replication set using SPSS.

## Figures and Tables

**Table 1:** Clinical and biochemical characteristics between the healthy and HTN groups in the discovery set.

	Total (*n* = 100)				*p^a^*	*p^b^*
	Healthy (*n* = 50)		HTN (*n* = 50)			
Age (year)	46.4	±0.76	53.2	±0.87	<0.001	**-**
Male/Female *n*, (%)	29 (58.0)/21 (42.0)		27 (54.0)/23 (46.0)		0.687	**-**
Body weight (kg)	68.1	±1.49	72.0	±1.59	0.074	0.999
BMI (kg/m^2^)	24.6	±0.40	26.6	±0.50	0.002	**-**
SBP (mmHg)	121.2	±1.52	134.2	±2.63	<0.001	0.001
DBP (mmHg)	75.0	±1.34	81.9	±1.31	<0.001	<0.001
Glucose (mg/dL)	95.1	±2.35	122.2	±5.89	<0.001	0.002
Triglyceride (mg/dL)	151.3	±14.2	133.3	±10.0	0.560	0.280
HDL-cholesterol (mg/dL)	52.3	±2.07	50.5	±1.72	0.551	0.836
LDL-cholesterol (mg/dL)	96.1	±5.62	102.2	±4.03	0.114	0.004
Total cholesterol (mg/dL)	194.9	±5.26	169.1	±4.91	0.003	0.076
AST (U/L)	21.1	±1.37	25.5	±1.26	0.013	0.041
ALT (U/L)	20.6	±1.57	27.4	±2.25	0.010	0.033
γ-GTP (U/L)	27.4	±3.57	39.7	±5.51	0.064	0.037

Values are presented as mean ± standard error (SE). All continuous variables were log-transformed prior to analysis. *p^a^*-Values for continuous variables were obtained using independent *t*-tests, while the *p^a^*-value for sex distribution was calculated using the chi-squared test. *p^b^*-Values represent *p^a^*-values adjusted for confounding factors, including age and BMI. Statistical significance was defined as *p* < 0.05. ALT: alanine aminotransferase. AST: aspartate aminotransferase. BMI: body mass index. DBP: diastolic blood pressure. γ-GTP: gamma-glutamyl transpeptidase. HDL: high-density lipoprotein. LDL: low-density lipoprotein. SBP: systolic blood pressure.

**Table 2:** LASSO regression analysis on major metabolites to select key metabolites for establishing MRS.

Independent Variables	LASSO Coefficient
2-Methylbutyrylcarnitine	−2.580545775
Choline	0
Creatine	0
Arginine	0
Pantothenic acid	0
Hexanoylcarnitine	0
Pyroglutamic acid	0
Phenylacetylglutamine	−0.112076792
Proline	0
1-Phenyl-1,3-octadecanedione	3.263081733
3-Indoxyl sulphate	0
4-Ethylphenylsulfonic acid	0
4-Phenolsulfonic acid	−0.32909885
Tryptophan	−1.176058849
Deoxycholic acid	−0.758505997
Nervonic acid	0.118245965
p-Cresylsulfate	−0.396155752
Trans-10-Heptadecenoic acid	0

Among the eighteen major metabolites (VIP ≥ 1 and FDR-adjusted *p*-value < 0.05), eight metabolites with non-zero LASSO coefficients were selected as key metabolites.

**Table 3:** Accuracy of re-grouping based on the MRS cut-off in the replication set.

MRS Re-Grouped Classification	Original Diagnosis		Total
	Healthy	Hypertension	
Healthy	42/50 (84.0%)	8/49 (16.3%)	50
Hypertension	8/50 (16.0%)	41/49 (83.7%)	49
Total	50	49	99

*p* < 0.001 (chi-squared test). Rows indicate the MRS cut-off-based re-grouped classification; columns indicate the original diagnosis in the replication set. Each cell reports *n*/N (%), where *n* is the number of individuals in each combination of MRS cut-off-based classification and original diagnosis, and N is the total number of individuals in that original-diagnosis column. Row and column totals represent the sums of the corresponding *n* values.

## Data Availability

The data that support the findings of this study are not openly available due to reasons of sensitivity and are available from the corresponding author upon reasonable request.

## Supplementary Materials

The following supporting information can be downloaded at: https://www.mdpi.com/article/10.3390/ijms27188130/s1.
