# Supplementary material for: A Molecular Diagnostic Approach for Hypertension Through Establishment of a Metabolite Risk Score Using a Multi-Metabolite Panel Identified via UHPLC-MS/MS
Source: Int J Mol Sci. 2026 Sep 12;27(18):8130. doi: 10.3390/ijms27188130 (PMC13607471; doi:10.3390/ijms27188130)
Supplement: Supplementary file 1 [file ijms-27-08130-s001.zip › Supplementary Figures (S1-S5).pdf]

(A) ESI Positive mode

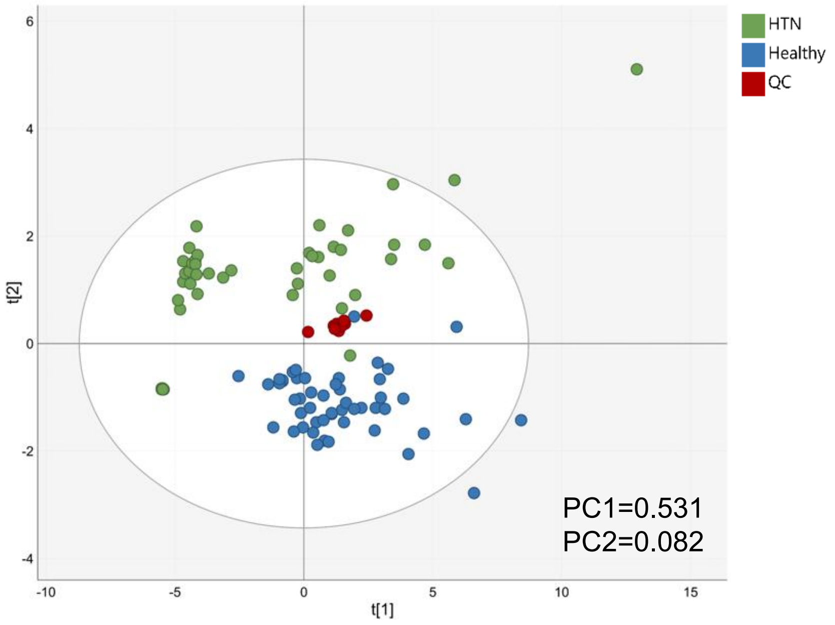

(B) ESI Negative mode

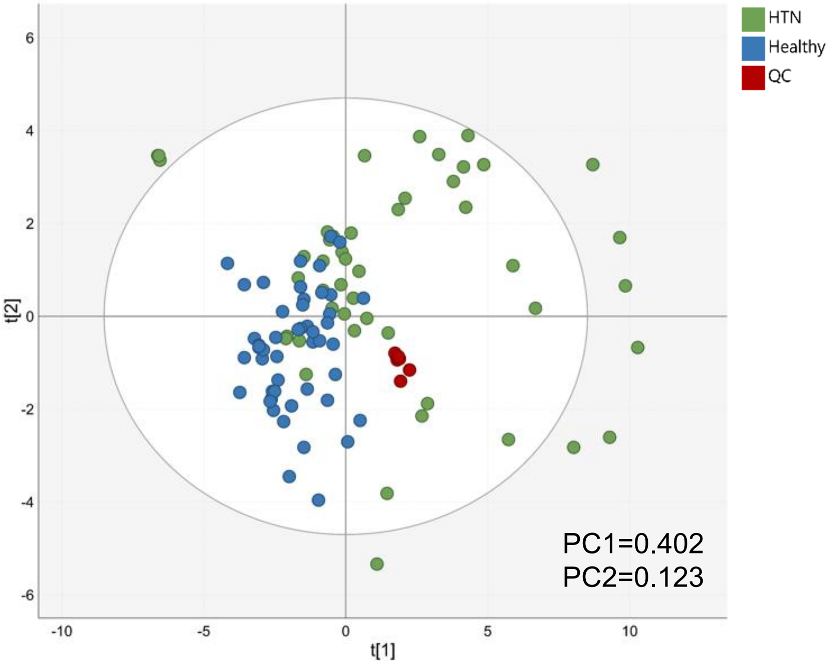

Figure S1. PCA score plots of plasma metabolic profiles in the discovery set.

(A) PCA score plot for ESI positive mode in the discovery set. The first two principal components explained 61.3% of total variance in the ESI positive mode (PC1=53.1%, PC2=8.2%). **(B)** PCA score plot for ESI negative mode in the discovery set. The first two principal components explained 52.5% of total variance in the ESI negative mode (PC1=40.2%, PC2=12.3%).

(A) Representative TIC of a pooled QC sample acquired in ESI positive mode

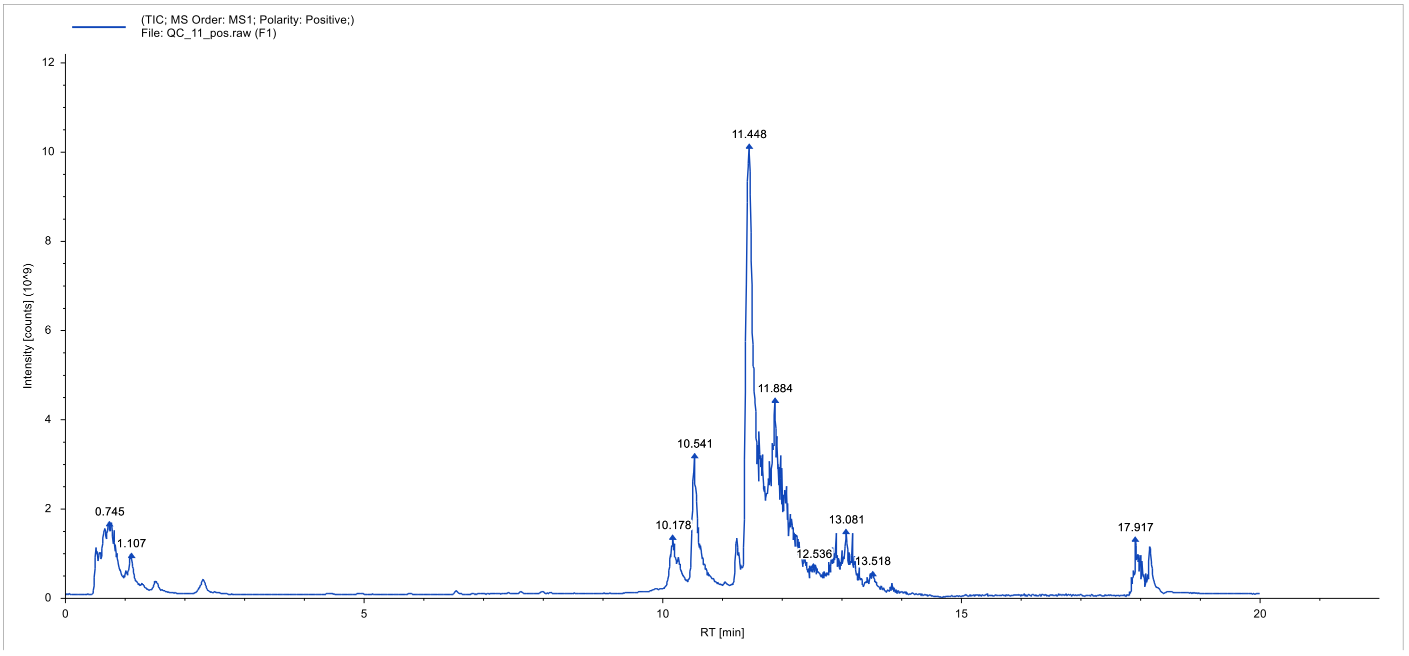

(B) Representative TIC of a pooled QC sample acquired in ESI negative mode

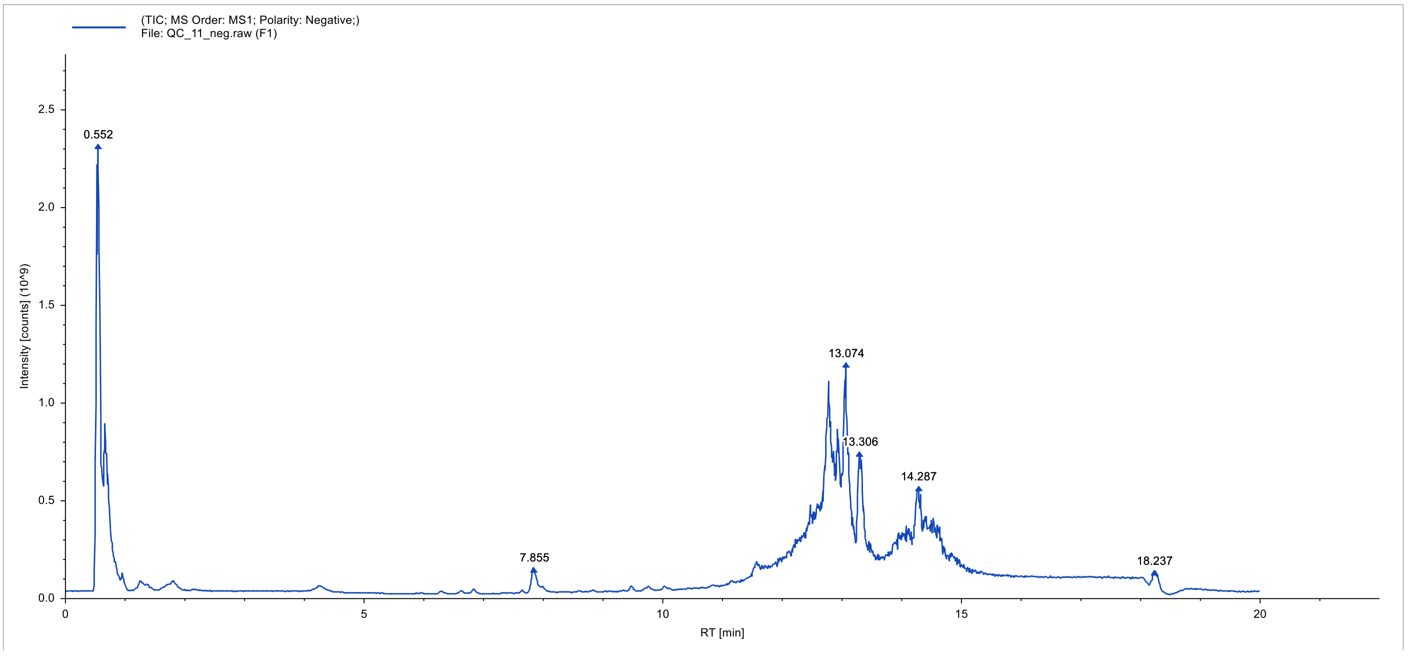

Figure S2. Representative TICs of pooled QC samples.

TICs were generated from representative pooled QC injections used during the UHPLC-MS/MS analysis. **(A)** ESI positive mode. **(B)** ESI negative mode.

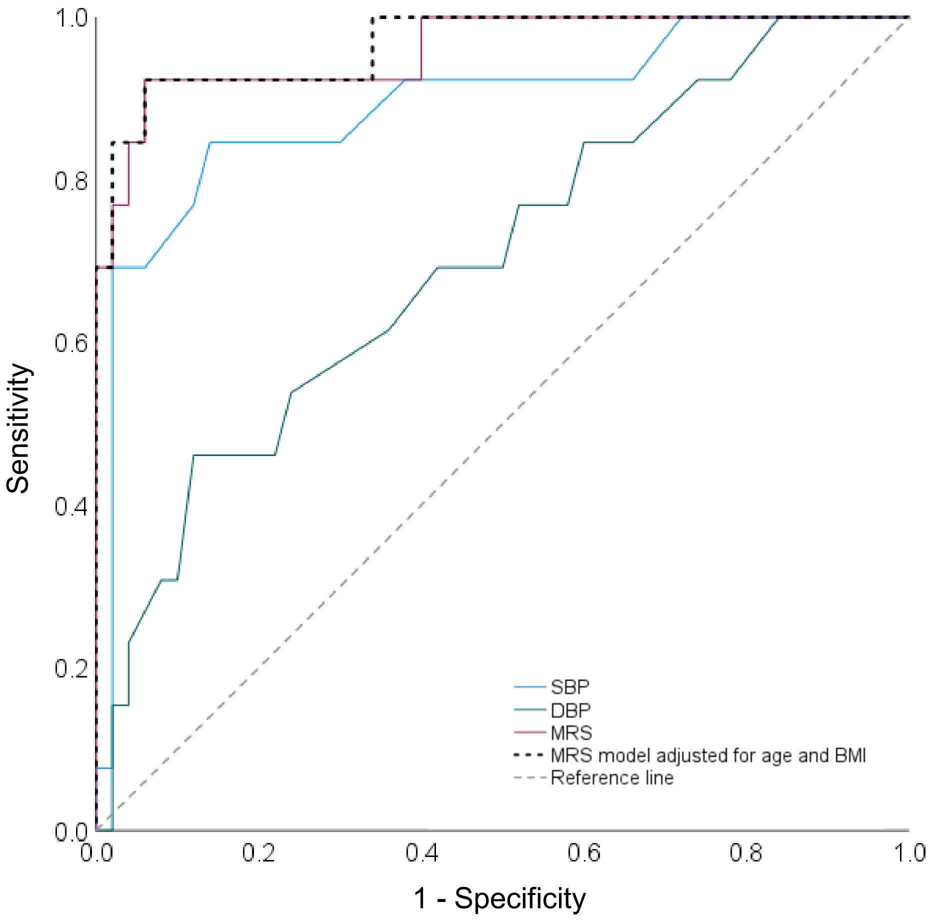

Model	AUC	SE	p	95% CI
SBP	0.892	0.057	<0.001	0.780–1.000
DBP	0.701	0.082	0.027	0.539–0.862
MRS	0.960	0.031	<0.001	0.899–1.000
MRS (adjusted)	0.966	0.027	<0.001	0.914–1.000

Figure S3. Sensitivity analysis of the discriminatory performance of the MRS compared with conventional blood pressure measures for prevalent hypertension in the replication set.

As the MRS was developed using the discovery set, the sensitivity analysis was conducted only in the replication set, which was designated for model evaluation. The analysis compared healthy individuals (n=50) with HTN participants without any recorded comorbid disease histories (n=13). The MRS continued to distinguish HTN from healthy participants after excluding those with recorded comorbidities, suggesting that the observed discrimination in the original analysis was not solely driven by comorbid conditions among participants with HTN.

(A) Discovery set

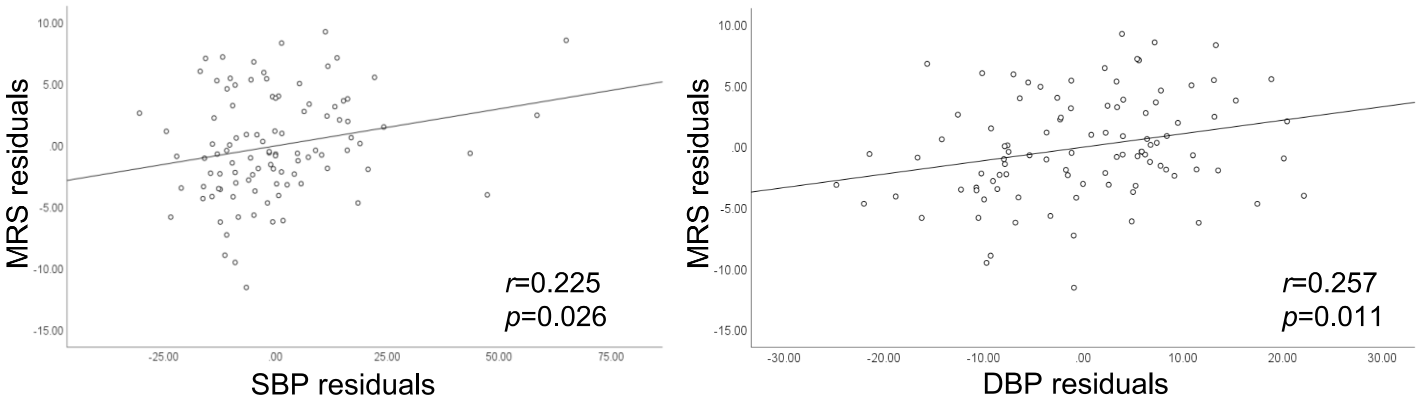

(B) Replication set

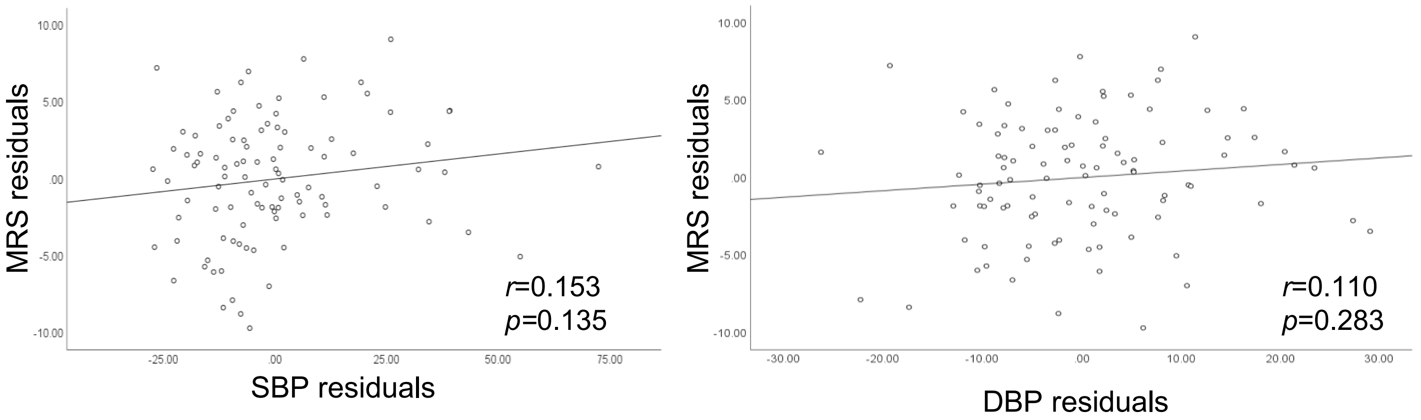

Figure S4. Age- and BMI-adjusted partial correlations between MRS and blood pressures in the discovery and replication sets.

Partial correlation analysis, which assesses the relationship between residuals after removing the effects of confounding factors, was performed to adjust for age and BMI. **(A)** Scatter plots of age- and BMI-adjusted residuals and partial correlation coefficients (*r*) between MRS and blood pressures (SBP and DBP) among all participants in the discovery set. **(B)** Scatter plots of age- and BMI-adjusted residuals and partial correlation coefficients (*r*) between MRS and blood pressures (SBP and DBP) among all participants in the replication set.

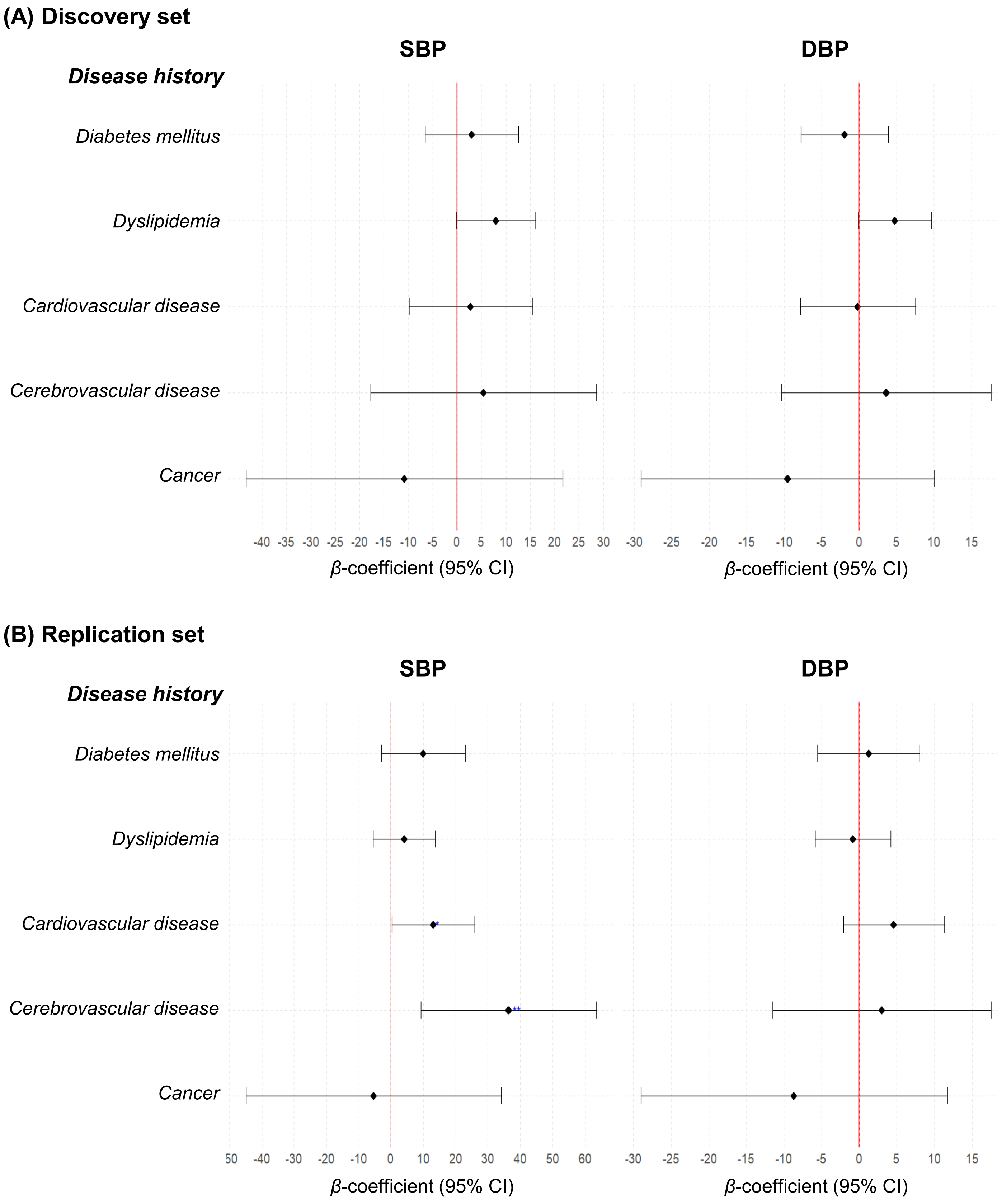

Figure S5. Association between blood pressures and disease history

Association between disease history and the conventional diagnostic factors (blood pressure) in the discovery and replication sets. No significant association was observed in the discovery set, whereas systolic blood pressure (SBP) was significantly higher in individuals with a history of cardiovascular and cerebrovascular diseases in the replication set. However, diastolic blood pressure (DBP) did not show any significant association with disease history in this set. $^{*}p < 0.05$, $^{**}p < 0.01$, and $^{***}p < 0.001$.
