# Supplementary material for: A Molecular Diagnostic Approach for Hypertension Through Establishment of a Metabolite Risk Score Using a Multi-Metabolite Panel Identified via UHPLC-MS/MS
Source: Int J Mol Sci. 2026 Sep 12;27(18):8130. doi: 10.3390/ijms27188130 (PMC13607471; doi:10.3390/ijms27188130)
Supplement: Supplementary file 1 [file ijms-27-08130-s001.zip › Supplementary Tables (S1-S2).pdf]

Table S1. Putatively identified metabolites

Putative metabolites	*m/z*	Discovery set		Replication set	
		Log$_2$ (fold change): HTN/healthy	*q*	Log$_2$ (fold change): HTN/healthy	*q*
**ESI positive mode**					
(2E)-3-(3-Hydroxyphenyl)acrylaldehyde	166.0859	-0.341	<0.001	-0.349	0.002
2-Methylbutyrylcarnitine	246.1698	-2.821	<0.001	-2.592	<0.001
3-(6,7-Dimethoxy-1,3-benzodioxol-5-yl)-2-propen-1-ol	239.0911	0.166	<0.001	0.725	0.006
3-Hydroxyoctanoylcarnitine	304.2116	-0.720	<0.001	-0.048	<0.001
4-Indolecarbaldehyde	146.0599	-0.515	<0.001	-0.412	<0.001
5-Hydroxyindole-3-acetic acid	192.0653	-0.590	<0.001	-0.223	<0.001
Acetyl-L-carnitine	204.1227	-1.564	<0.001	-1.593	<0.001
Asp-Leu	247.1286	-0.989	<0.001	-0.608	<0.001
Betaine	118.0861	-0.803	<0.001	-0.855	<0.001
Choline	104.1068	-0.833	<0.001	-0.487	<0.001
Creatine	132.0766	-0.747	<0.001	-0.073	<0.001
Creatinine	114.066	-1.285	<0.001	0.042	<0.001
Proline	116.0704	-1.015	<0.001	-0.808	<0.001
Arginine	175.1188	-1.555	<0.001	-1.110	<0.001
Carnitine	162.1123	-0.205	<0.001	-0.063	0.009
Pantothenic acid	220.1177	-1.529	<0.001	-0.464	<0.001
Ethyl argininate	203.1499	-1.259	<0.001	-0.746	<0.001
Hexanoylcarnitine	260.1854	-1.837	<0.001	-1.228	<0.001

Leucine	132.1017	0.777	0.023	0.783	0.152
Pyroglutamic acid	130.0497	-1.243	<0.001	-0.970	<0.001
Phenylacetylglutamine	265.1179	-1.385	<0.001	1.335	<0.001
Propionylcarnitine	218.1384	0.083	<0.001	0.352	0.001
Trans-3-Indoleacrylic acid	188.0703	-0.362	<0.001	0.121	0.018
**ESI negative mode**					
10-Undecenoic acid	183.1391	0.427	0.058	0.602	0.021
Eicosadienoic acid	307.2643	0.990	0.046	0.413	0.085
Eicosenoic acid	309.28	1.334	0.027	0.941	0.205
1-Palmitoyl-2-hydroxy-sn-glycero-3-PE	452.2778	-0.255	0.006	-0.495	0.001
1-Phenyl-1,3-octadecanedione	357.2792	1.708	<0.001	1.236	0.088
3-Indoxyl sulphate	212.0023	-0.378	<0.001	0.256	0.015
4-Ethylphenylsulfonic acid	201.0228	0.122	<0.001	-0.207	0.002
4-Phenolsulfonic acid	172.9913	-0.293	0.003	-0.014	0.003
4-Vinylphenol sulfate	199.0072	0.720	0.058	0.440	0.055
Docosapentaenoic acid	329.2486	1.318	0.018	0.906	0.259
Androsterone sulfate	369.1734	-0.292	0.099	-0.157	0.961
Arachidonic acid	303.233	0.663	0.138	0.255	0.018
Tryptophan	203.0826	-0.093	0.010	-0.245	0.003
Deoxycholic acid	391.2846	-0.464	0.001	-0.087	0.021
Docosahexaenoic acid	327.2329	1.088	0.103	0.808	0.077
Eicosapentaenoic acid	301.2173	1.277	0.025	1.106	0.363

Glycocholic acid	464.3015	0.453	0.021	-0.807	0.025
Guaiacol sulfate	203.002	0.535	0.026	0.316	0.227
Isocitric acid	191.0196	-0.060	0.052	-0.437	0.001
Lauric acid	199.1704	0.524	<0.001	1.132	0.006
Linoelaidic acid	279.2328	1.106	0.135	0.590	0.041
Tyrosine	180.0667	0.312	0.040	0.088	0.786
Nervonic acid	365.3419	1.019	0.003	0.733	0.802
Oleic acid	281.2484	1.304	0.054	0.773	0.106
Palmitoleic Acid	253.2173	1.341	0.044	0.783	0.106
p-Cresylsulfate	187.007	-0.644	<0.001	-0.079	0.014
Taurochenodeoxycholic acid	498.2893	0.872	0.085	-0.781	0.017
Trans-10-Heptadecenoic acid	267.233	1.343	<0.001	0.965	0.786
Uric acid	167.0211	-0.062	0.013	-0.095	0.008

Differences between healthy and HTN groups in each dataset were evaluated using independent t-tests with false discovery rate (FDR) correction for Type I error. Statistical significance was defined as an FDR-adjusted *q*-value <0.05.

Table S2. Association between the MRS and prevalent HTN in the replication set

Outcome	Analysis	Model	Coefficient *β*	Odds ratio (OR)	95% Confidential Interval (CI)	*p*
HTN	Primary replication analysis†	MRS (crude)	0.637	1.890	1.462 – 2.445	<0.001
		MRS (adjusted)	0.558	1.747	1.317 – 2.318	<0.001
	Sensitivity analysis§	MRS (crude)	1.065	2.900	1.349 – 6.232	0.006
		MRS (adjusted)	0.980	2.665	1.100 – 6.461	0.030

†Primary replication analysis: all participants in the replication set (healthy n=50, HTN n=49). §Sensitivity analysis: healthy individuals (*n*=50) and HTN participants without recorded comorbid disease histories (*n*=13) in the replication set. Logistic regression was performed to assess whether the associations between the MRS and prevalent HTN were retained after accounting for potential confounding by age and BMI. The crude model included MRS only, whereas the adjusted model additionally included age and BMI as covariates. In both primary and sensitivity analyses, the MRS remained significantly associated with HTN after adjustment for age and BMI, indicating that the observed associations were not fully accounted for by differences in age and BMI.
